# Some Laundry Statistics

**Published:** 1908-07-11

**Authors:** 


### Nursing Administration.

### SOME LAUNDRY STATISTICS.

The reports which are now being issued by hospitals in the Metropolitan area for 1907 are of unusual interest to all interested in hospital management. It has been recognised by the Council of the King's Fund that the first and most vital step towards economy of management in institutions is to be fully acquainted with the cost and results of working different systems, and to this end they have invited from such hospitals as desire to be included in the grants made by the fund detailed returns of expense incurred under certain headings. We have for many years preached this doctrine of comparative finance, and the principal drawback to its consistent application has hitherto been the difficulties which lie in the way of obtaining serviceable statistics. One of the bars to comparing expenditure between one institution and another was abolished by the introduction of the uniform system of accounts, and wherever that is in use the managers have a ready means of checking their ordinary outgoings by comparison with other hospitals of similar size and circumstances. A gradual change is observable in the administration of some of the larger establishments. There is an increasing tendency to make the institution self-sufficing in a number of directions, and wherever this is done there is danger that unremunerative enterprises may be maintained under a delusive belief that economy is promoted, unless the accounts of each branch of the work undertaken be carefully disentangled from the whole and presented in such a form that it may appear whether the business is carried on successfully or no. In regard to the laundry, statistics have been sadly to seek. Under the head of washing the widest variation existed as to the items included when the work was carried on in premises attached to the hospital, and it has been practically impossible for any inquirer to ascertain what financial benefit, if any, a hospital might look to obtain by inaugurating a private laundry. Last yeaı, however, the King's Fund asked for a washing return which should define exactly the position of the private hospital laundry from the point of view of expenditure. It does not by any means follow that the hospital is bound to take the cheapest course in respect of the washing. The advantages of having a private laundry are overwhelming from sanitary and administrative reasons. But it is a matter of great importance that the laundry should be managed on business lines, and the only way to ensure that this is done is to compare the sums spent in this direction by one hospital with another, and to compare both with the sums spent on washing by hospitals which contract for the work to be done off the premises, in a manner entirely outside their control. We append tables showing the cost under various items of washing done on and off the hospital premises in certain hospitals.

We feel bound to point out that the figures which appear to work out against the private laundry are rather heavily weighted by the amount written off under the head of "depreciation." This amounts to £1 per bed in the case of Guy's and to £2 per bed in the case of the Middlesex Hospital, and although, to the auditors' mind, these charges are indispensable, they are hardly expenditure in the sense of being money paid out. It must also be taken into consideration that the hospital laundry sends the linen back to the ward sorted, aired, and in many cases mended, whereas the outside laundry can seldom be depended on to this extent, and hence entails additional expense over and above the washing bill. The disinfection and preliminary purification of foul linen, too, is in the case of the private laundry carried out by the laundry staff, even if conducted in a separate building, and some arrangement has to be made for this when the washing is put out. In general it may be taken for granted that the laundry work, when carried out under the direct superintendence of the hospital authorities, is conducted on altogether superior lines to those of the ordinary commercial laundry. When the enormous importance of cleanliness in all the processes connected with the bedding of the hospital is borne in mind, it does not seem that even the addition of as much as a pound or two per bed to the cost should be considered too high a price to pay for the hospital laundry.

**Washing Returns, 1907.—On the Premises.**

| | Guy's | Middle sex | Poplar | Royal Free |
|---|---|---|---|---|
| Average number of occupied beds | 515 | 314 | 77 | 138 |
| (a) Salaries, wages | £1,978 | £675 | £204 | £350 |
| (b) Maintenance of laundry hands | — | — | 109 | — |
| Wages of workers whose time is wholly or partly given to laundry, engineers, &c. | 136 | 252 | 92 | 100 |
| Materials and Sundries (soap, soda, brooms, &c., baskets, overalls) ... | 354 | 122 | 90 | 95 |
| Fuel, power and light .................. | 624 | 328 | 119 | 250 |
| Water .......................................... | 40 | 116 | 16 | 35 |
| Repairs to (a) Laundry building | 371 | 61 | 5 | 50 |
| (b) Machinery & plant | | 60 | 35 | |
| Insurance—Fire and boiler............ | 46 | 4 | 7 | nil |
| Depreciation—Buildings and Machinery .................................. | 549 | 601 | 31 | 80 |
| Interest on capital ......................... | 746 | 480 | 48 | 100 |
| Rates ........................................... | 189 | 107 | ½ | nil |
| Carriage....................................... | nil | 69 | nil | nil |
| Total cost of washing .................. | 4,746 | 2,881 | 760 | 1,060 |
| Average weekly number of pieces washed .................................. | 21,856 | 16,539 | 3,071 | 4,500 |
| Annual amount of work done......... | 1,078,746 pieces | 860,028 pieces | 159,692 pieces | 234,000 pieces |
| Cost of washing per occupied bed... | £9 | £9 | £10 | £7 |

**Washing Returns, 1907.—Off the Premises.**

| | St. Thomas's | St. George's | King's College Hospital | Homœopathic Hospital | Hampstead General Hospital | Miller Hospital |
|---|---|---|---|---|---|---|
| Average of occupied beds.................... | 480 | 319 | 189 | 83 | 46 | 22 |
| Annual cost of washing................. | £3,049 | £2,697 | £1,346 | £553 | £329 | £232 |
| Average weekly number of pieces washed ............... | 16,712 | 12,847 | 8,218 | 1,928 | 1,506 | 958 |
| Yearly amount of work done (pieces) | 869,024 | 667,940 | 427,336 | 100,250 | 78,312 | 49,816 |
| Cost of washing per occupied bed ....... | £6¼ | £8 | £7 | £6½ | £7 | £10½ |

